# Novel RNA Virus Genome Discovered in Ghost Ants (*Tapinoma melanocephalum*) from Hawaii

**DOI:** 10.1128/genomeA.00669-17

**Published:** 2017-07-27

**Authors:** Laura E. Brettell, Gideon J. Mordecai, Purnima Pachori, Stephen J. Martin

**Affiliations:** a School of Environment and Life Sciences, University of Salford, Manchester, United Kingdom; b Viral Ecology, Marine Biological Association, Plymouth, United Kingdom; c Earlham Institute, Norwich, United Kingdom

## Abstract

Here, we report the full-genome sequence of Milolii virus, a novel single-stranded (positive-sense) RNA virus discovered from *Tapinoma melanocephalum* ants in Hawaii. The genome is 10,475 nucleotides long, encoding a polyprotein of 3,304 amino acids.

## GENOME ANNOUNCEMENT

The ghost ant (*Tapinoma melanocephalum* [Fabricius]) is a widely distributed invasive pest, probably of African or Asian origin (1), that is now found in tropical and subtropical climates as well as in glass houses in temperate regions (2). The species was first recorded in Hawaii in 1899 (3) and soon became a common household pest (4).

Ghost ants are now a major pest across the Pacific Islands (5) and Florida (6) and are listed in the Global Invasive Species Database (7). They are highly adaptable, feeding on waste food (2), and in hospitals, they pose an additional threat of carrying pathogenic bacteria (8).

The combination of an invasive species with an emerging disease poses a potentially major risk to biodiversity (9), since many RNA viral pathogens have wide host ranges that can belong to different but overlapping food webs. This provides vast potential for viral pathogens to spread via different networks to new hosts (10, 11).

RNA was extracted from two pooled samples of 20 to 25 *T. melanocephalum* ants collected from inside two honey bee (*Apis mellifera*) hives from an apiary in Milolii, Big Island, Hawaii, in December 2012, using the RNeasy minikit (Qiagen). Oligo(dT) priming was used to create cDNA libraries, which were sequenced using the Illumina HiSeq 2000 platform at the Earlham Institute, Norwich, United Kingdom (formerly the Genome Analysis Centre), to produce 100-bp paired-end reads. Quality filtering was carried out using FastQC. Kontamination, a pipeline developed by TGAC to remove host reads, was applied using the related reference *Linepithema humile* ant genome due to the unavailability of a *T. melanocephalum* genome. The resulting reads were pooled and IVA version 1.0.3 (12) was used to generate *de novo* assembled contigs, which resulted in the assembly of the Milolii virus genome.

The genome is 10,475 nucleotides (nt) long with a 3′ polyadenylated tail end and a 9,930-nt open reading frame encoding a polyprotein of 3,304 amino acids. A BLASTx search of the protein-coding sequences against the NCBI protein database revealed conserved RNA helicase and RNA-dependent RNA polymerase (RdRp) domains typical of single-stranded (positive) RNA viruses. The protein-coding sequences showed closest matches (29% to 30%) to the hypothetical protein of Hubei Tetragnatha maxillosa virus 5 (13) and the P1 protein of Acrythosiphon pisum virus (14), both invertebrate viruses. The coverages across the protein-coding sequences were 56% and 60%, respectively. For each sample, total reads (FASTA format) were mapped against the Milolii virus genome in Geneious (Biomatters). Both showed full-length genome coverages with depths ranging from approximately 1,000× to 1,000,000× (data not shown), with 28.12% and 69.65% of the total reads aligning to Milolii virus. Furthermore, full-length genomes were assembled individually from each of the two samples, and Geneious nucleotide alignments revealed that the sequences share 99.9% pairwise nucleotide similarity.

### Accession number(s).

This full-genome sequence has been submitted to GenBank under the accession number MF155030.
